# HMGCS2 Mediates Ketone Production and Regulates the Proliferation and Metastasis of Hepatocellular Carcinoma

**DOI:** 10.3390/cancers11121876

**Published:** 2019-11-26

**Authors:** Yuan-Hsi Wang, Chao-Lien Liu, Wan-Chun Chiu, Yuh-Ching Twu, Yi-Jen Liao

**Affiliations:** 1 School of Medical Laboratory Science and Biotechnology, College of Medical Science and Technology, Taipei Medical University, Taipei 110, Taiwan; m609105001@tmu.edu.tw (Y.-H.W.); chaolien@tmu.edu.tw (C.-L.L.); 2 Department of Biotechnology and Laboratory Science in Medicine, National Yang-Ming University, Taipei 112, Taiwan; yctwu@ym.edu.tw; 3 School of Nutrition and Health Sciences, Taipei Medical University, Taipei 110, Taiwan; wanchun@tmu.edu.tw; 4 Research Center of Geriatric Nutrition, College of Nutrition, Taipei Medical University, Taipei 110, Taiwan

**Keywords:** Hepatocellular carcinoma, HMGCS2, ketone body

## Abstract

Hepatocellular carcinoma (HCC) is the most common primary malignant tumor worldwide; however, the traditional therapeutic approaches and survival rates are still limited. To improve current therapies, it is necessary to investigate the molecular mechanisms underlying liver cancer and to identify potential therapeutic targets. The aims of this study were to verify the mechanisms and therapeutic potential of the ketogenesis rate-limiting enzyme 3-Hydroxymethylglutaryl-CoA synthase 2 (HMGCS2) in HCC. Immunohistochemical staining of human liver disease tissue arrays showed that HMGCS2 is abundantly expressed in normal liver tissues but is downregulated in cirrhosis and HCC tissues. In HCC patients, lower HMGCS2 expression was correlated with higher pathological grades and clinical stages. In our investigation of the molecular mechanisms of HMGCS2 in HCC, we showed that knockdown of HMGCS2 decreased ketone production, which promoted cell proliferation, cell migration, and xenograft tumorigenesis by enhancing c-Myc/cyclinD1 and EMT signaling and by suppressing the caspase-dependent apoptosis pathway. Ketone body treatment reduced the proliferation- and migration-promoting effects of HMGCS2 knockdown in cells. In contrast, HMGCS2 overexpression increased the intracellular ketone level and inhibited cell proliferation, cell migration, and xenograft tumorigenesis. Finally, ketogenic diet administration significantly inhibited liver cancer cell growth in mice. Our studies highlight the potential therapeutic strategy of targeting HMGCS2-mediated ketogenesis in liver cancer.

## 1. Introduction

Relative to normal cells, cancer cells exhibit significant alterations in metabolism. To satisfy the increasing energy demand, cancer cells exhibit an atypical metabolic phenotype of increased glycolysis and lactate metabolism [1,2,3]. In the absence of glucose, cellular energy is produced mainly from the degradation of fatty acids. Fatty acids are catabolized in the liver and undergo β-oxidation to produce acetyl-CoA. Once the acetyl-CoA flux exceeds the capacity of citrate synthesis, the surplus acetyl-CoA becomes the substrate for ketogenesis and generates ketone bodies. In contrast to normal cells, cancer cells have difficulty shifting their energy source from glucose to ketone bodies but prefer to use alternative fuel sources, including acetate, glutamine, and aspartate [4,5]. Since ketone metabolism is a nutrient response, it is worth studying its biomedical connections to cancer due to the potential for nutritional therapy.

Primary liver cancer is the seventh leading cause of cancer death worldwide, and hepatocellular carcinoma (HCC) accounts for nearly 85% of all primary liver cancer cases [6]. Patients with HCC have a poor prognosis, and the five-year survival rate remains low at 16.6% [7]. HCC is often diagnosed at an intermediate or advanced stage, and thus, the effectiveness of HCC treatment is often limited [8]. Recently, metabolism has provided another approach for the treatment of HCC [9]. The alterations in metabolic function in HCC include gluconeogenesis, fatty acid oxidation, lipid utilization, and ketogenesis [10,11]. In particular, the connections between ketone bodies and cancer progression are rapidly emerging. However, studies in both animal models and humans have yielded diverse conclusions in different cancer types. A previous study showed that breast cancer cells are able to induce D-β-hydroxybutyrate (βHB) production in adjacent fibroblasts and to furnish tumor cells with energy to grow and metastasize [12,13]. In the liver, hepatoma cells shift from ketogenesis to ketone utilization [14]. Conversely, stimulating the production of βHB in melanoma and glioblastoma cells causes tumor cell growth arrest [15]. In pancreatic cancer cells, treatment with ketone bodies inhibits growth, proliferation, and glycolysis [16]. Poff et al. also found similar results, indicating that ketone supplementation inhibits the proliferation of metastatic glioblastoma cells [17]. Collectively, these findings indicate that the roles of ketone body metabolism differ among cancer types. Since most ketone production occurs in the liver, revealing the effect of ketone bodies in liver cancer is necessary.

3-Hydroxymethyl glutaryl-CoA synthase 2 (HMGCS2) is the rate-limiting enzyme in the ketogenesis pathway [18]. HMGCS2 gene expression is under transcriptional regulation by PPARα, which upregulates genes involved in fatty acid oxidation and ketogenesis [19,20]. During fasting, the surplus acetyl-CoA becomes the substrate for ketogenesis. Two molecules of acetyl-CoA condense to form acetoacetyl-CoA (AcAc-CoA) in a reaction catalyzed by acetoacetyl-CoA thiolase. Next, HMGCS2 catalyzes the condensation of AcAc-CoA and acetyl-CoA to generate hydroxymethylglutaryl-CoA (HMG-CoA). HMG-CoA is then converted into the first type of ketone body, acetoacetate (AcAc), and into acetyl-CoA by HMG-CoA lyase. The majority of acetoacetate is then reduced to βHB by β-hydroxybutyrate dehydrogenase, and the remaining AcAc is spontaneously decarboxylated into acetone [21,22]. The expression of HMGCS2 has been found in normal liver, skeletal muscle, heart, pancreas, testis, and colon tissue [23]. In human cancer, the expression of HMGCS2 is different. For example, increased HMGCS2 expression was found in estrogen receptor-negative breast cancer and apocrine carcinoma of the breast [24,25]. In poorly differentiated colon cancer, HMGCS2 protein expression was downregulated [26]. High expression of HMGCS2 caused poor susceptibility of rectal cancer to chemoradiotherapy [27]. Although HMGCS2 expression was decreased in hepatoma cell lines and hepatocellular carcinoma patients [28,29], the regulation of ketogenesis by HMGCS2 remains unclear in HCC.

In this study, we provided new insights into the mechanisms linking HMGCS2 downregulation-induced defects in ketone production with liver cancer cell proliferation and migration. Regulating HMGCS2-mediated ketone production in HCC may therefore represent a new treatment strategy for liver cancer.

## 2. Results

### 2.1. HMGCS2 Expression Is Decreased in Liver Cancer Tissues

**Figure 1:**
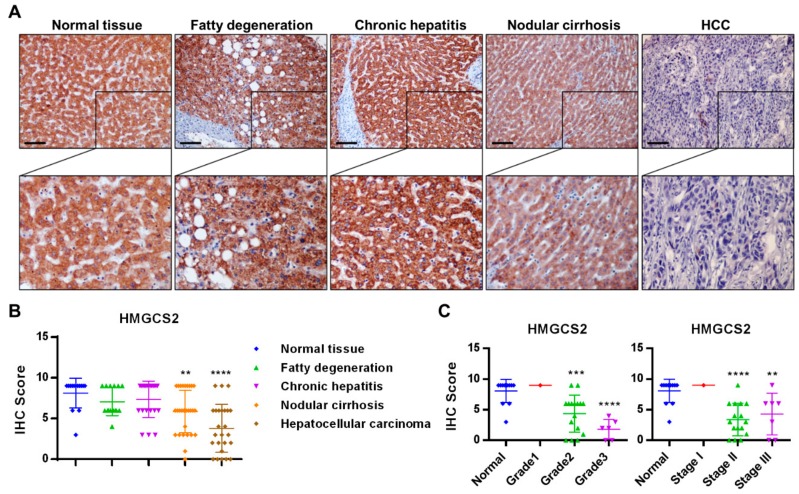
HMGCS2 (3-Hydroxymethylglutaryl-CoA synthase 2) expression is decreased in hepatocellular carcinoma (HCC) liver tissues. (**A**) HMGCS2 expression was detected by immunohistochemical (IHC) staining. The black bar represents a length of 0.1 mm. (**B,C**) The IHC scores were calculated by multiplying the two scores of the proportion of positively stained area and the immunostaining intensity. Normal tissue, *n* = 14; fatty degeneration, *n* = 15; chronic hepatitis, *n* = 22; nodular cirrhosis, *n* = 30; HCC, *n* = 25 (grade 1, *n* = 1; grade 2, *n* = 16; grade 3, *n* = 6. Two samples without grading information were excluded. Stage I, *n* = 1; stage II, *n* = 16; stage III, *n* = 7. One sample classified as stage Ivb was excluded.) ** *p* < 0.01; *** *p* < 0.001; **** *p* < 0.0001 vs. blue line. Data are shown as mean ± SD.

To reveal the expression profiles of HMGCS2 during the development of HCC, we used a liver tissue array to compare HMGCS2 expression among normal tissues and tissues representing fatty degeneration, chronic hepatitis, nodular cirrhosis, and liver cancer. The results of immunohistochemical (IHC) staining demonstrated that HMGCS2 mainly localized at the cytoplasm and that HMGCS2 expression was decreased in nodular cirrhosis and significantly reduced in HCC tissues, while the fatty degeneration and chronic hepatitis liver tissues showed no change compared with the normal liver tissues (Figure 1A,B). In addition, the expression of HMGCS2 was significantly decreased in tissues from higher pathological grades and clinical stages of HCC (Figure 1C). The collective data obtained from the clinical human specimens indicated that HMGCS2 protein expression is negatively correlated with disease progression and severity of hepatocellular carcinoma.

### 2.2. Establishment of Cell Lines with Stable HMGCS2 Overexpression and Knockdown

**Figure 2:**
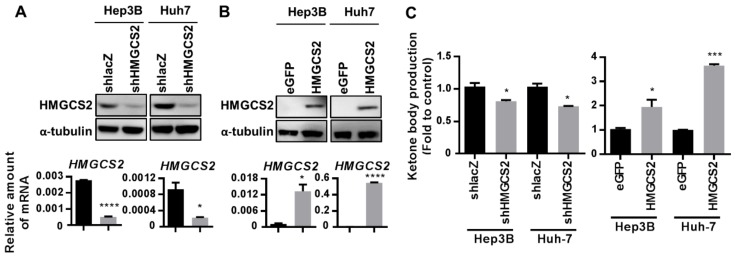
Establishment of cell lines with stable HMGCS2 overexpression and knockdown: (**A,B**) Western blotting and QPCR were used to assess HMGCS2 gene expression in shHMGCS2 and HMGCS2-overexpressing cells. (**C**) Ketone production in both HMGCS2 knockdown and HMGCS2-overexpressing Hep3B and Huh-7 cells was confirmed with a colorimetric βHB assay kit. *, *p* < 0.05; *** *p* < 0.001; **** *p* < 0.0001 vs. black bar. Data are shown as mean ± SD.

To clarify the roles of HMGCS2 expression in liver cancer cells, the HMGCS2 gene was either knocked down or overexpressed in Hep3B and Huh-7 cells by lentivirus infection. Puromycin (1 μg/mL) was added to the culture medium to select the gene-transfected stable cells. HMGCS2 protein and mRNA expression in the Hep3B and Huh-7 cell lines were verified by western blotting and quantitative real-time polymerase chain reaction (QPCR) assays, respectively (Figure 2A,B). Protein quantification was performed by using the ImageJ system (Supplementary Figure S1). Functional ketogenesis activity in both HMGCS2 knockdown and HMGCS2-overexpressing cells was confirmed by a colorimetric βHB assay (Figure 2C). There were no significant morphological changes between the different HMGCS2 gene-modified cells (Supplementary Figure S2). These data indicated that the HMGCS2 overexpression and knockdown cell lines were successfully established and may functionally reflect ketogenesis activity.

### 2.3. Genes and Biological Functions Affected by Downregulated Expression of HMGCS2 in HCC Cells

**Figure 3:**
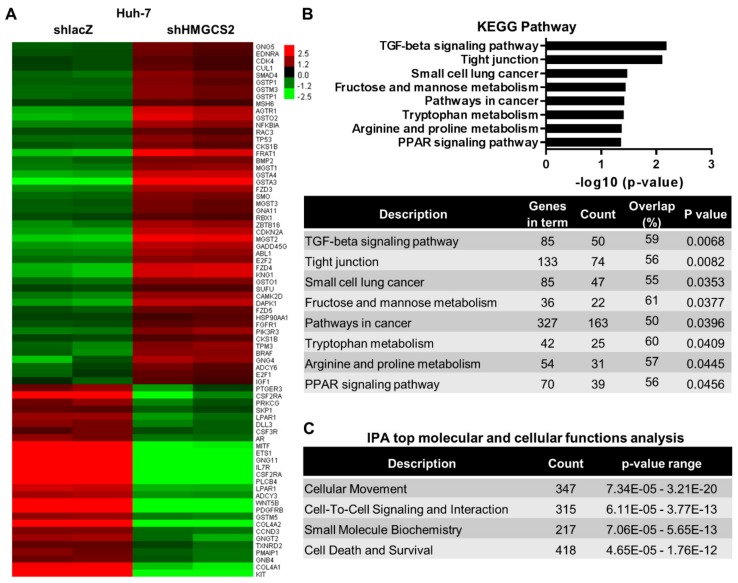
Gene expression profile of Huh-7 HMGCS2 knockdown cells: (**A**) The heat map of differentially expressed genes in Huh-7 shlacZ and shHMGCS2 cells based on mRNA microarray analysis. A significant difference was observed in mRNA expression between control and HMGCS2 knockdown cells. (**B**) Kyoto Encyclopedia of Genes and Genomes (KEGG) pathway enrichment analysis for up- and downregulated genes between normal and HMGCS2 knockdown cells. (**C**) Top molecular and cellular functions classified by Ingenuity Pathway Analysis (IPA), with the corresponding number of molecules after HMGCS2 knockdown in Huh-7 cells.

To analyze alterations in the gene expression profile, total RNA was extracted from Huh-7 shlacZ and shHMGCS2 cells for microarray experiments. In microarray analysis, a 2-fold increase or decrease in the signal intensity is considered a significant change in mRNA expression (Figure 3A). To reveal the pathways dysregulated by HMGCS2 gene knockdown, Kyoto Encyclopedia of Genes and Genomes (KEGG) pathway enrichment analysis was performed. The results demonstrated that the dysregulated genes in Huh-7 shHMGCS2 cells were enriched in pathways related to cancer progression, including TGF-β signaling pathway, tight junction, and pathways in cancer (Figure 3B; Supplementary Figures S3–S5), which implied the possibility that HMGCS2 controls cancer cell motility and growth. The Ingenuity Pathway Analysis (IPA) package was used to detect enriched molecular and cellular functions. Cellular movement, cell-to-cell signaling and interaction, and cell death and survival were markedly changed after HMGCS2 gene knockdown (Figure 3C). In addition, the expression of genes related to ERK/MAPK signaling, PI3K/AKT signaling, EMT pathway, and molecular mechanism of cancer pathways were also identified by using IPA (Supplementary Figures S6–S9). 

### 2.4. HMGCS2 Suppresses Liver Cancer Cell Growth by Increasing Apoptosis and Inhibiting Proliferation

**Figure 4:**
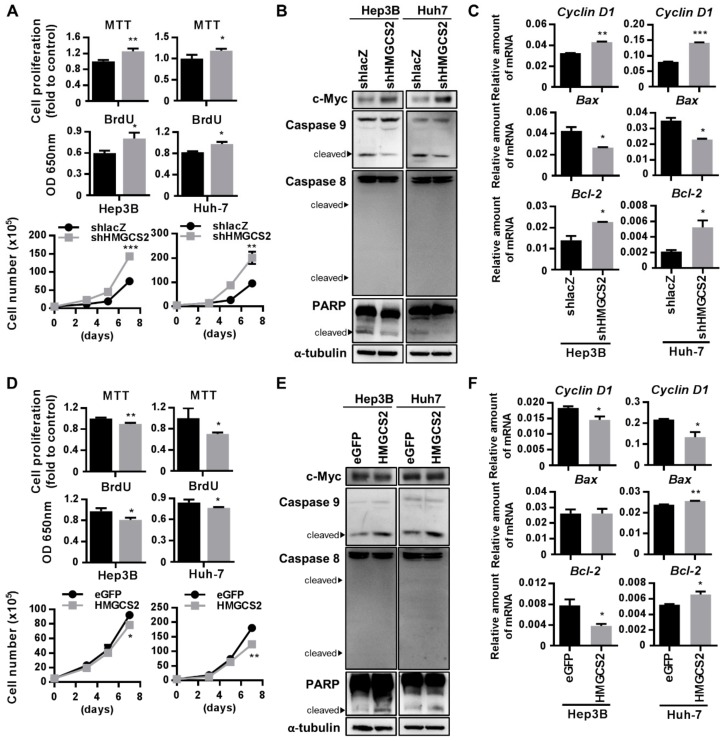
HMGCS2 expression influences cells proliferation and apoptosis. (**A,D**) Different gene-modified cells (3 × 10^3^ cells/well) were seeded in a 96-well plate, and the proliferation was analyzed by MTT assay and BrdU assay after 48 h. Trypan blue cell viability assay was also used to analyze the cell proliferation. Different gene-modified cells (5 × 10^4^ cells/well) were seeded in a 6-well plate, and the cell number was analyzed for 7 days. (**B,E**) Protein expression of caspase 9, caspase 8, PARP, and c-Myc in different gene-modified cells were analyzed by the western blot. (**C,F**) mRNA expression of Bax, Bcl-2, and cyclin-D1 in different gene-modified cells were analyzed by QPCR. * *p* < 0.05; ** *p* < 0.01; *** *p* < 0.001 vs. black bar. Data are shown as mean ± SD.

The biological functions of HMGCS2 in the progression of HCC were next investigated. The results of the 3-(4,5-Dimethylthiazol-2-yl)-2,5-diphenyltetrazolium bromide (MTT) assay, 5-bromo-2′-deoxyuridine (BrdU) assay, and cell counting showed that knockdown of HMGCS2 increased cell growth (Figure 4A). On the other hand, overexpression of HMGCS2 in both Hep3B and Huh-7 cells inhibited cell growth (Figure 4D). C-Myc and cyclin D1 are important markers of cell proliferation; thus, the protein expression of c-Myc and mRNA expression of cyclin D1 were also analyzed. The results showed that knockdown of HMGCS2 in Hep3B and Huh-7 cells enhanced c-Myc and cyclinD1 expression (Figure 4B,C), while overexpression of HMGCS2 did not change c-Myc expression but decreased cyclinD1 expression (Figure 4E,F). Since cell apoptosis is another factor that influences cell growth; the mRNA expressions of Bax and Bcl-2 and the protein expressions of caspase 9, caspase 8, and PARP were investigated. As expected, the protein expression levels of cleaved PARP and caspase 9 were decreased in the shHMGCS2 group, and the level of cleaved caspase 8 did not differ between the cell types (Figure 4B). The mRNA expression of Bax was decreased, while that of Bcl-2 was increased in both Hep3B and Huh-7 shHMGCS2 cells compared with control cells (Figure 4C). On the other hand, HMGCS2-overexpressing cells showed increased cleavage of PARP and caspase 9 and unchanged cleavage of caspase 8 (Figure 4E). Bax mRNA expression was unchanged in Hep3B cells but slightly increased in Huh-7 shHMGCS2 cells, while Bcl-2 mRNA expression was decreased in Hep3B cells but increased in Huh-7 shHMGCS2 cells (Figure 4F). The quantification of all western blot protein data was performed with the ImageJ system (Supplementary Figures S10 and S11). These data demonstrated that HMGCS2 expression is correlated with HCC cell proliferation and apoptosis through the regulation of c-Myc/cyclin D1 and caspase-dependent signaling.

### 2.5. Low Expression of HMGCS2 Is Correlated with Enhanced Migration Ability

**Figure 5:**
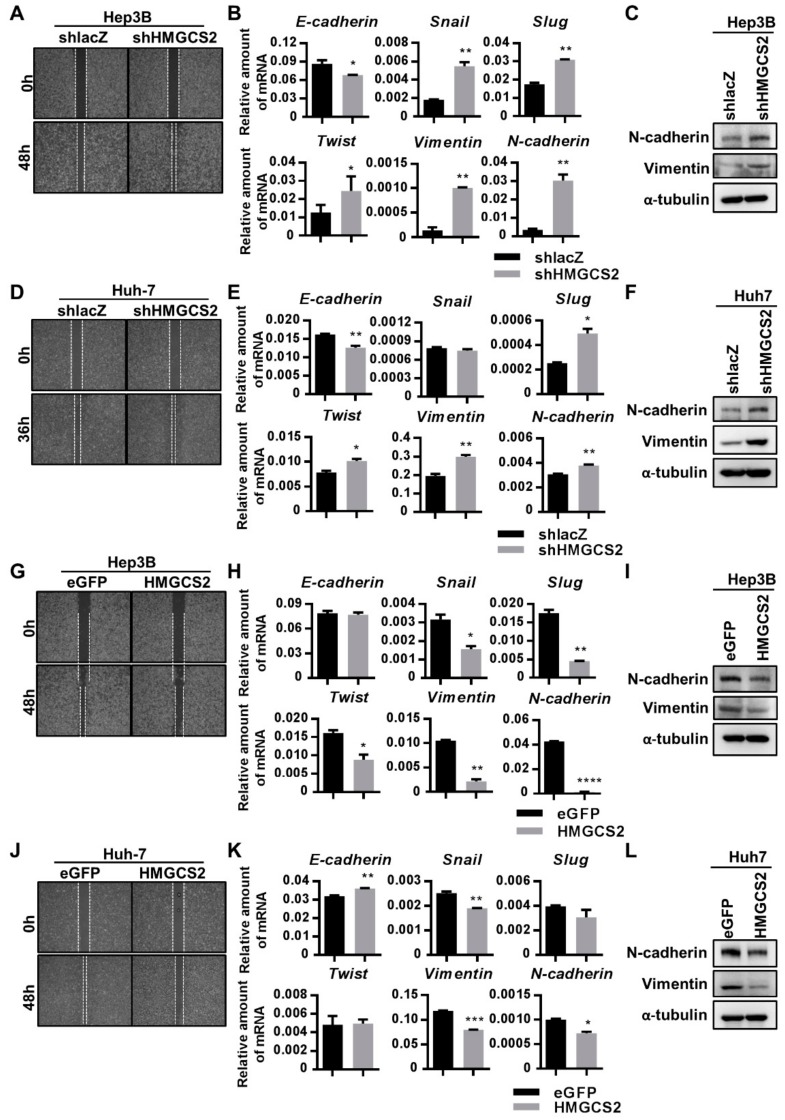
HMGCS2 expression regulates cell migration and EMT-related marker expression. (**A,D**) HMGCS2 downregulation and (**G,J**) overexpression of Hep3B and Huh-7 cells (4 × 10^5^ cells/well) were seeded in ibidi Culture-Insert dishes; the cell migration was documented with a digital camera. (**B,E,H,K**) Gene expression of E-cadherin, Snail, Slug, Twist, Vimentin, and N-cadherin were analyzed by using QPCR. (**C,F,I,L**) Protein expression of N-cadherin and Vimentin were analyzed by western blot. * *p* < 0.05; ** *p* < 0.01; *** *p* < 0.001; **** *p* < 0.0001 vs. black bar. Data are shown as mean ± SD.

To explore whether HMGCS2 expression influences the migration ability of HCC cells, a wound healing assay was conducted and epithelial-mesenchymal transition (EMT)-related markers were analyzed. The results showed that knockdown of HMGCS2 in both Hep3B and Huh-7 cells significantly enhanced the cell migration ability (Figure 5A,D). In addition, the mRNA expression of E-cadherin was reduced in both Hep3B and Huh-7 shHMGCS2 cells (Figure 5B,E). The expression levels of Snail, Slug, Twist, Vimentin, and N-cadherin were significantly elevated in Hep3B shHMGCS2 cells compared with shlacZ control cells (Figure 5B). In addition, Huh-7 shHMGCS2 cells showed enhanced Slug, Twist, Vimentin, and N-cadherin mRNA expression (Figure 5E). Moreover, the protein expression levels of N-cadherin and Vimentin were significantly elevated in HMGCS2 knockdown cells (Figure 5C,F). Protein quantification was performed by using the ImageJ system (Supplementary Figure S12). On the other hand, both Hep3B and Huh-7 cells with HMGCS2 overexpression showed a reduced cell migration ability (Figure 5G,J). The mRNA expression of Snail, Slug, Twist, Vimentin, and N-cadherin was significantly decreased in HMGCS2-overexpressing Hep3B cells (Figure 5H). In Huh-7 HMGCS2 cells, the mRNA expression of E-cadherin was increased and that of Snail, Vimentin, and N-cadherin was decreased (Figure 5K). The protein expression of N-cadherin and Vimentin was significantly reduced in both Hep3B and Huh-7 shHMGCS2 cells (Figure 5I,L). Protein quantification was performed using the ImageJ system (Supplementary Figure S12). Based on these findings, we concluded that HMGCS2 expression influences the cell migration ability through regulation of the EMT pathway.

### 2.6. Ketone Body Supplementation Reduces the Increase in the Proliferation and Migration Abilities of HMGCS2 Knockdown Cells

**Figure 6:**
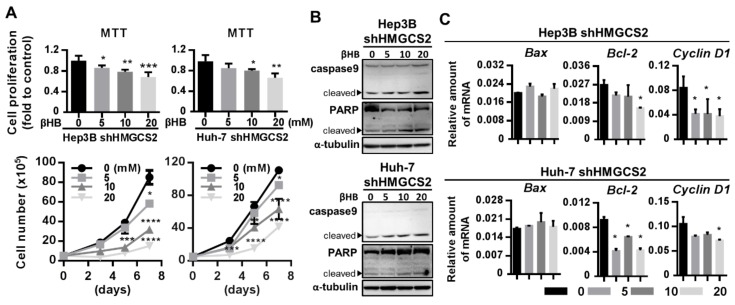
Ketone body supplement reduces the higher proliferative effects in HMGCS2 knockdown cells. (**A**) Hep3B and Huh-7 shHMGCS2 cells were seeded in a 96-well plate (3 × 10^3^ cells/well) and a 6-well plate (5 × 10^4^ cells/well). Different concentration of βHB (0, 5, 10, and 20 mM) were added, and the cell proliferation was analyzed by MTT assay and trypan blue cell viability assay. (**B**) HMGCS2 knockdown of Hep3B and Huh-7 were treated with or without 5 mM βHB for 24 h, and the protein expressions of caspase9 and PARP were analyzed by the western blot. (**C**) Hep3B and Huh-7 shHMGCS2 were treated with or without 5 mM βHB for 24 h, and the mRNA expression of Bax, Bcl-2, and cyclin-D1 were analyzed by QPCR. * *p* < 0.05; ** *p* < 0.01; *** *p* < 0.001; **** *p* < 0.0001 vs. black bar. Data are shown as mean ± SD.

Since HMGCS2 is the rate-limiting gene in the ketone body production pathway [18], we further investigated whether HMGCS2 gene expression influences HCC progression in a ketone-dependent manner. The MTT assay and cell counting results showed that treatment of both Hep3B and Huh-7 shHMGCS2 cells with different concentrations of βHB significantly inhibited cell growth in a dose-dependent manner (Figure 6A). The western blot results showed that the levels of the cleaved forms of PARP and caspase 9 were also elevated after βHB treatment in both Hep3B and Huh-7 shHMGCS2 cells (Figure 6B). Protein quantification was performed by using the ImageJ system (Supplementary Figure S13). After shHMGCS2 cells were treated with βHB, the mRNA expression of Bax was unchanged and the expression of Bcl-2 was decreased (Figure 6C). In addition, the QPCR data showed that the expression of cyclin D1 was reduced after βHB treatment in both lines of HMGCS2 knockdown cells (Figure 6C).

**Figure 7:**
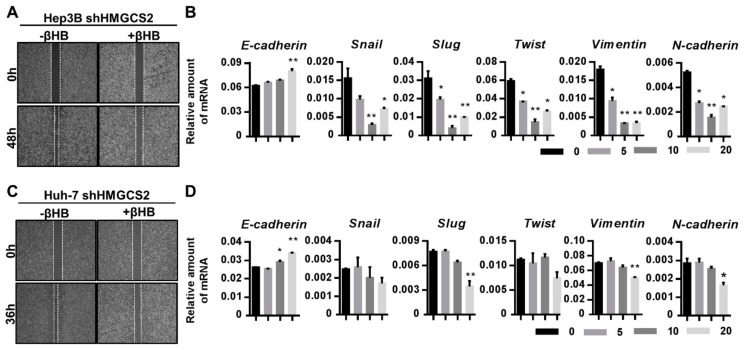
Ketone body supplement rescues the higher migration effects in HMGCS2 knockdown cells. (**A,C**) The wound-healing assay was used to determine cell migration. Hep3B and Huh-7 shHMGCS2 cells (4 × 10^5^ cells/well) were seeded in ibidi Culture-Insert dishes and treated with 5 mM βHB for 48 and 36 h, respectively. Wound closure was documented with a digital camera. (**B,D**) HMGCS2-knockdown Hep3B and Huh-7 cells were treated with or without 5 mM βHB for 24 h, and the gene expression of E-cadherin, Snail, Slug, Twist, Vimentin, and N-cadherin was analyzed by using QPCR. * *p* < 0.05; ** *p* < 0.01 vs. black bar. Data are shown as mean ± SD.

A wound healing assay was conducted, and EMT-related markers were analyzed. These results showed that βHB-treated HMGCS2 knockdown cells exhibited a reduced migration ability compared with untreated control cells (Figure 7A,C). The mRNA expression of E-cadherin was increased in both lines of βHB-treated HMGCS2 knockdown cells (Figure 7B,D). In Hep3B shHMGCS2 cells, the mRNA expression of Snail, Slug, Twist, Vimentin, and N-cadherin was reduced after treatment with βHB (Figure 7B). In Huh-7 shHMGCS2 cells, treatment with βHB inhibited the mRNA expression of Slug, Vimentin, and N-cadherin, while the Snail and Twist expression levels were also decreased, although the difference was not statistically significant (Figure 7D). These results demonstrated that HMGCS2 gene expression regulates HCC cell proliferation and migration in a ketone-dependent manner. In addition, ketone body supplementation reduced the proliferation and migration ability of HMGCS2 knockdown liver cancer cells.

### 2.7. HMGCS2-Related Ketone Production Affects HCC Tumor Growth In Vivo

**Figure 8:**
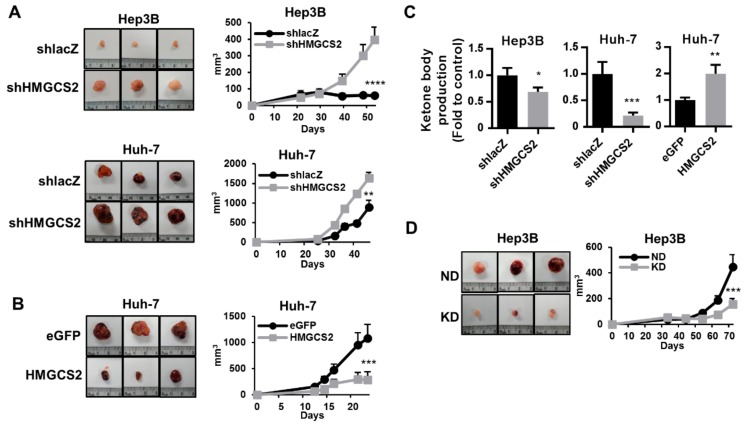
HMGCS2 expression influences the tumor growth in vivo. (**A,B**) Hep3B (shlacZ and shHMGCS2) and Huh-7 (eGFP and HMGCS2) cells (5 × 10^6^) were injected subcutaneously into the NOD/SCID mice to measure the tumor growth in vivo. (**C**) The ketone production in the tumor mass was analyzed by a colorimetric β-hydroxybutyrate assay kit. (**D**) The ketogenic diet was given one week before Hep3B cells were injected subcutaneously into the NOD/SCID mice and tumor growth in vivo was measured. * *p* < 0.05; ** *p* < 0.01; *** *p* < 0.001; **** *p* < 0.0001 vs. black line. Data are shown as mean ± SD.

To determine whether the HMGCS2 expression level is related to tumor growth, in vivo xenograft models were used in further experiments. HCC cells were subcutaneously implanted into NOD/SCID mice, and the tumor size was measured. The results showed that knockdown of HMGCS2 enhanced tumor formation (Figure 8A). On the other hand, HMGCS2-overexpressing Huh-7 cells showed a lower tumor formation ability than control cells (Figure 8B). To determine whether the influence of HMGCS2 on tumor formation is correlated with the level of ketone bodies, the concentration of ketone bodies in the tumor mass was evaluated. The results showed that shHMGCS2 tumors had decreased concentrations of ketone bodies (Figure 8C). However, in HMGCS2-overexpressing tumors, the concentration of ketone bodies was higher than that in control tumors (Figure 8C). These results demonstrated that the HMGCS2-regulated ketone body concentration in tumors is a key factor that influences tumor growth. Thus, a ketogenic diet was further used to evaluate the antitumor effect of such a diet in liver cancer. The results showed that mice fed the ketogenic diet had a significantly lower subcutaneous tumor growth rate than mice in the normal diet group (Figure 8D).

## 3. Discussion

**Figure 9:**
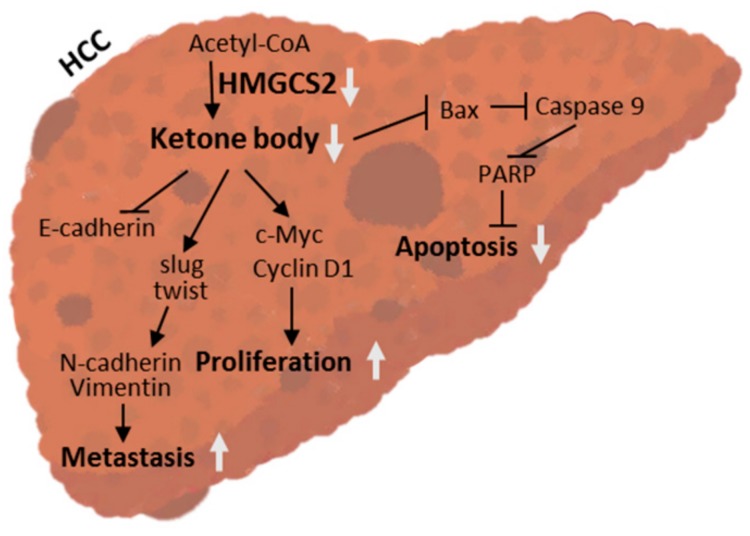
HMGCS2-mediated ketogenesis impacts liver cancer progression and treatment. The mechanism operates as follows: Downregulation of HMGCS2 expression reduces ketone production and promotes liver cancer cell proliferation and migration by enhancing c-Myc/cyclin D1 and EMT signaling and by suppressing the caspase-dependent apoptosis pathway. Administration of a ketogenic diet significantly inhibited liver cancer cell growth in mice. These findings highlight the potential therapeutic strategy of targeting HMGCS2-mediated ketogenesis in liver cancer.

Hepatocellular carcinoma is one of the most common malignancies worldwide, and its incidence and mortality continue to increase annually [30,31]. Recently, the metabolic distinction between HCC cells and normal hepatocytes has provided a new strategy for targeting cancer cells [1,2]. The identification of new biomarkers with potent prognostic or therapeutic value may benefit HCC patients. In this study, we demonstrated that decreased HMGCS2 expression was correlated with the severity of HCC, that cell proliferation was promoted via the regulation of the intrinsic apoptosis and c-Myc/cyclin D1 pathways, and that cell migration was enhanced by upregulating the EMT signaling pathway. HMGCS2 knockdown HCC cells showed enhanced tumor growth in a xenograft mouse model. Ketone supplementation was confirmed to significantly reduce cell proliferation and migration abilities in a ketone concentration-dependent manner; the ketogenic diet significantly inhibited subcutaneous tumor growth (Figure 9).

Relative to normal cells, cancer cells possess an unusual energy metabolism phenotype [32]. HMGCS2, the gene that regulates ketone body production, generates lipid-derived energy in liver cells [33]. Under normal circumstances, HMGCS2 is silenced in proliferating cells but is detectable in differentiated cells [28,34,35]. In colorectal adenocarcinoma, HMGCS2 expression was downregulated in moderately and poorly differentiated carcinomas, which implied the role of HMGCS2 in the regulation of tumor differentiation [26]. Our data supported the hypothesis that low expression of HMGCS2 is closely associated with poor HCC differentiation (Figure 1). In addition, HMGCS2 knockdown in HCC cells inhibited cell proliferation (Figure 4). The effect of HMGCS2 on cell proliferation was supported by previous research showing that fenofibrate-induced HMGCS2 upregulation in melanoma cells was accompanied by proliferation arrest [15]. On the other hand, knockdown of HMGCS2 in HCC cells markedly promoted cell migration (Figure 5). Su et al. showed that miR107-mediated HMGCS2 downregulation enhanced the migration ability of HepG2 cells [29]. Martinez-Outschoorn and colleagues reported that HMGCS2 overexpression endowed MDA-MB-231 cells with metastatic ability [12]. Thus, in addition to hormonal and metabolic signals, HMGCS2 plays an important role in the regulation of cell proliferation and migration. Furthermore, HMGCS2 is a gene that controls ketone body production in the liver under physiological conditions, and decreased HMGCS2 protein levels are correlated with decreased enzyme activity [18,36,37]. Our data supported the concept that modifying the expression of this gene in HCC cells induces a relative increase or decrease in ketone production (Figure 2). To verify whether the HMGCS2 gene influences HCC progression in a ketone-dependent manner, cells were treated with the downstream metabolic products of HMGCS2. Notably, serum levels of ketone bodies vary in different situations; two days of fasting can increase the ketone body concentration to 1–2 µM [38,39], prolonged starvation can cause an increase to 6–8 µM [40], and in pathological states, the ketone body concentration can rise as high as 20 µM [22]. Thus, we used 0, 5, 10, and 20 µM βHB in the study. These ketone body concentrations have also been applied in many other published articles [16,41,42,43]. Our data demonstrated that ketone body supplementation reduces the increased proliferation and migration of HMGCS2 knockdown cells (Figure 6; Figure 7). In addition, the cell apoptosis pathway was significantly activated and the mRNA expression of EMT-related markers was repressed after ketone body supplementation (Figure 6 and Figure 7). Previous research showed that ketone supplementation decreases tumor cell viability and inhibits metastatic cancer cell proliferation [17]. Treatment with ketone bodies inhibited growth and induced apoptosis in pancreatic cancer cell lines [16]. Therefore, our findings suggest the novel concept that ketogenesis appears to be involved in HMGCS2-promoted HCC progression.

The regulation of HMGCS2 expression occurs mainly at the transcriptional level. It has been reported that the HMGCS2 gene contains a peroxisome proliferator response element (PPRE) in the promoter region [44]. HMGCS2 interacts with PPARα and acts as a coactivator to upregulate the transcription of its own gene [44,45]. In addition, this interaction is enhanced by palmitoylation of HMGCS2 on Cys-166 [44]. Interestingly, we found that Huh-7 shHMGCS2 cells exhibited a 3.4-fold increase in PPARα expression relative to that in control cells in the mRNA microarray analysis. In the liver, PPARα acts as a master regulator of liver metabolism. In previous studies, the role of PPARα in tumorigenesis has been widely studied. However, some studies implicated PPARα in the promotion of cancer while others presented evidence for an antitumorigenic role [46]. Recently, Xiao et al. found that high PPARα expression was correlated with prolonged survival times in HCC patients [47]. From these results, it can be inferred that, although PPARα is the upstream molecule that promotes HMGCS2 expression, HMGCS2 plays a more important role than PPARα in affecting HCC cell progression. Camarero et al. found that, in addition to PPARα, HMGCS2 is a direct target of c-Myc that represses HMGCS2 transcriptional activity [26]. In our study, we found that knockdown of HMGCS2 increased c-Myc expression while overexpression of HMGCS2 showed unchanged c-Myc protein expression (Figure 4B,E). In addition, Huh-7 shHMGCS2 cells exhibited a 2.3-fold increase in Myc expression relative to that in control cells in the mRNA microarray analysis (data not shown). Since it has been reported that HMGCS2 expression is not related to the c-Myc level and that only some tumors are Myc dependent [48], the HMGCS2 and c-Myc expression levels may depend on additional factors related to the nutritional status or intestinal microflora of patients [26,48].

Recent studies have provided evidence that HMGCS2 showed altered expression levels and had prognostic implications in different human cancers. For example, HMGCS2 overexpression is a negative prognostic factor in breast cancer, rectal cancer, and prostate cancer [24,27,49]. On the other hand, HMGCS2 downregulation indicates poor outcomes in esophageal squamous cell carcinoma and colon cancer [26,48]. In the liver tissue microarray analysis in our study, IHC staining of HMGCS2 demonstrated that HMGCS2 expression was slightly decreased in nodular cirrhosis tissue and significantly reduced in HCC liver tissue while fatty degeneration and chronic hepatitis liver tissue showed no change compared with normal liver tissue (Figure 1). Moreover, the expression of HMGCS2 was negatively correlated with the pathological grade and clinical stage (Figure 1). These data suggested that HMGCS2 serves as a promising prognostic biomarker and exerts antitumor activity in HCC. This concept was supported by our animal experimental data. In the in vivo mouse model (Figure 8), we found that HCC cells with HMGCS2 downregulation showed an increased tumor growth ability. In contrast, tumors formed from HMGCS2-overexpressing HCC cells showed a decreased growth rate. Quantification of the ketone body concentration indicated that HMGCS2-regulated tumor growth occurs in a ketone-dependent manner (Figure 8C). Recently, the connection between ketone bodies and cancer has been considered as a new strategy for treating cancer; the application of ketogenic diets as therapeutic tools has also emerged. A ketogenic diet consists of a high fat content with a moderate protein content and a very low carbohydrate content, which forces the body to use fat instead of glucose for ATP synthesis [50]. The relationship between dietary lipids and cancer has been widely discussed [51]. Previous research found that gastric cancer growth in the ketogenic diet group was significantly delayed compared to that in the standard diet group [52]. Compared with a low-fat/high-carbohydrate diet, a ketogenic diet prolonged survival in a mouse model of prostate cancer [53]. However, the antitumor effect of a ketogenic diet on liver cancer remains unclear. In this study, we found that the ketogenic diet inhibited the growth of subcutaneously implanted HCC cells in NOD/SCID mice (Figure 8). This result was supported by a previous study that diethylnitrosamine (DEN)-treated female mice fed with the ketogenic diet had no tumor incidence [54]. Although the detailed mechanism by which the ketogenic diet inhibited HCC tumor growth requires further investigation, our data provide new insight into HMGCS2-mediated ketone production as a nutritional therapy in the treatment of hepatocellular carcinoma.

## 4. Materials and Methods

### 4.1. Immunohistochemical Staining

Human HCC tissue microarrays (LV1201) were purchased from US BioMax, Inc. (Ijamsville, MD, USA). Clinical and pathological information for the individual samples were obtained from the array manufacturer. Tissue slides with human HCC tissue microarrays were rehydrated. For antigen retrieval, slides were boiled in Target Retrieval Solution (DakoCytomation, Carpinteria, CA, USA) for 30 min in a water bath. Liver sections were incubated with the antibody and detected using a Universal LSABTM2 kit (DakoCytomation Carpinteria, CA, USA) according to the manufacturer’s instructions. The primary antibody (anti-HMGCS2, Abcam, Cambridge, MA, USA) (1:100) was applied and incubated at room temperature for 1.5 h. Finally, the signals were visualized and the slides were counterstained with hematoxylin. Each sample was scored by multiplying two scores: (1) the score for the proportion of positively stained area (0%, 0; 1–25%, 1; 26–50%, 2; 51–75%, 3; and 76%–100%, 4) and (2) the score for the immunostaining intensity (0, no staining; 1, weak staining; 2, mild staining; and 3, strong staining). The final score ranged from 0–9.

### 4.2. Cell Culture, Plasmids, and Lentivirus Infection

The 293T cell line was purchased from American Type Culture Collection (ATCC, No. CRL-11268) and cultured in Dulbecco’s modified Eagle’s medium (DMEM; Gibco BRL, Grand Island, NY, USA) supplemented with 10% fetal bovine serum (FBS; HyClone, Logan, UT, USA), penicillin and streptomycin (100 U/mL), nonessential amino acids (0.1 mM), and L-glutamine (2 mM) at 37 °C in a 5% CO_2_ incubator. Gene knockdown experiments used RNA interference; the plasmids contained the HMGCS2 shRNA (pLKO.1-shHMGCS2) and control lacZ shRNA (pLKO.1-shlacZ) sequences. For the overexpression experiments, the plasmid (pLKO_AS3w.eGFP.puro) was obtained from the National RNAi Core Facility (Academia Sinica, Taipei, Taiwan). TurboFect Reagent (Fermentas, Hanover, MD, USA) was used for co-transfecting the packaging plasmid, pCMV-DR8.91, obtained from RNAi Core Facility (Academia Sinica, Taipei, Taiwan), the VSV-G envelope expression plasmid (pMD.G), and one of four lentiviral vector constructs (pLKO.1-shlacZ, pLKO.1-shHMGCS2, pLKO_AS3w.eGFP.puro, or pLV-HMGCS2) into 293T cells. Supernatants containing lentiviruses were collected. Hep3B (purchased from BCRC, No. 60434) and Huh-7 [55] cells were infected with lentivirus in medium containing polybrene (8 μg/mL) for the generation of stable cell lines. After 24 h of infection, 1 μg/mL puromycin was added to select the stable cells. Gene-transfected stable cells (Hep3B/Huh-7 shlacZ, shHMGCS2, eGFP and HMGCS2) were cultured in DMEM supplemented with 10% FBS and 1 μg/mL puromycin (Sigma Aldrich, St Louis, MO, USA).

### 4.3. Colorimetric β-Hydroxybutyrate Assay

To evaluate the ketogenic activity of HMGCS2 overexpression and knockdown cell lines, ketone body production was analyzed by using a commercial Colorimetric β-Hydroxybutyrate Assay Kit (BioVision, Mountain View, CA, USA). The cell lysate (1 × 10^6^ cells/well) was collected, and the protein component was removed using a 10 Kd spin column (BioVision, Mountain View, CA, USA). Fifty microliters of the prepared sample was added to the commercial enzyme and substrate mix in a 96-well plate. After 30 min of incubation at room temperature with protection from light, the absorbance at 450 nm was evaluated to quantitatively measure the production of ketone bodies in cultured cells.

### 4.4. Trypan Blue Cell Viability Assay

Different gene-modified cells (5 × 10^4^ cells/well) were seeded in a 6-well plate. After serum starvation for 16 h, fresh 10% FBS DMEM was added and incubated for 7 days. The cell pellet was suspended in 1 mL of phosphate buffered saline (PBS) mixed 1:1 with 0.4% trypan blue (Gibco BRL, Grand Island, NY, USA). A hemacytometer (Hausser Scientific, Horsham, PA, USA) was used to count the cells.

### 4.5. MTT Cell Viability Assay

Different gene-modified cells (3 × 10^3^ cells/well) were seeded in a 96-well plate. After serum starvation for 16 h, fresh 10% FBS DMEM was added and incubated for 48 h. The culture medium was removed, and 50 μL of 1× MTT reagent (Sigma-Aldrich, St. Louis, MO, USA) was added and incubated for 3 h at 37 °C in a 5% CO_2_ incubator (Panasonic Healthcare Co, Tokyo, Japan). After incubation, 100 μL of dimethyl sulfoxide (Scharlab Chemie, Barcelona, Spain) was added and the absorbance at 570 nm was evaluated to quantitatively measure the proliferation of cultured cells.

### 4.6. BrdU Cell Proliferation Assay

Different gene-modified HCC cells (3 × 10^3^ cells/well) were seeded in a 96-well plate. After serum starvation for 16 h, fresh 10% FBS DMEM was added and incubated for 48 h. The cells were then incubated in BrdU solution (BioVision, Mountain View, CA, USA) for 2.5 h at 37 °C in a 5% CO_2_ incubator. The cells were then treated with fixing and denaturing solution for 30 min, BrdU detection antibody for 1 h, and anti-mouse horseradish peroxidase (HRP)-linked antibody for 1 h at room temperature. The level of cell proliferation was determined by measuring the absorbance at 450 nm.

### 4.7. Wound-Healing Migration Assay

Cell migration was assessed by using a wound-healing assay. Different gene-modified cells (4 × 10^5^ cells/well) were seeded in ibidi Culture-Insert dishes (ibidi GmbH, Martinsried, Germany) for 24 h at 37 °C in a 5% CO_2_ incubator. The culture inserts were gently removed by using sterile tweezers and were then filled with fresh culture medium.

### 4.8. Western Blot Analysis

Isolation of total protein from cultured cells was performed in lysis buffer supplemented with protease and phosphatase inhibitors. The protein concentration was measured by a protein assay (Bio-Rad Laboratories, CA, USA), and the amount of protein in all samples was normalized to 30 μg. Cellular proteins were separated by SDS-PAGE and transferred to polyvinylidene difluoride (PVDF) membranes. Primary and secondary antibodies were used to detect the targets on the membrane. The HMGCS2 antibody used in this study was purchased from Abcam (Cambridge, MA, USA). Antibodies against the following proteins were purchased from Cell Signaling (Beverly, MA, USA): caspase-9, caspase-8, PARP, and c-myc. The immunoreaction signals were normalized to those of α-tubulin (Sigma-Aldrich, St. Louis, MO, USA). The bands were visualized using enhanced chemiluminescence (ECL) detection reagent (Millipore Corporation, Billerica, MA, USA). Each result of the data presented in each figure was performed in three independent replicates. The similar phenomenon was observed, and therefore, representative data was shown in the figure. The ImageJ software (National Institutes of Health, Bethesda, MD, USA) was used to quantify the images.

### 4.9. Real-Time PCR (QPCR)

Total RNA was isolated from cultured cells with TRIzol Reagent (Ambion, Carlsbad, CA, USA) according to the manufacturer’s protocol. High-Capacity cDNA Reverse Transcription Kits (Applied Biosystems, Carlsbad, CA, USA) were used to produce complementary DNA from cellular RNA (2 μg). The template cDNA (20 ng in 4 µL), 5 µL of KAPA SYBR^®^ FAST qPCR Master Mix (2×), and 1 µL of forward/reverse primer mix (6 µM each) (KAPA Biosystems, Boston, MA, USA) were added to 48-well PCR plates for the reactions (10 μL). The thermal cycling program consisted of 15 min at 95 °C followed by 40 cycles at 95 °C for 15 s and 60 °C for 60 s in a StepOne system (Applied Biosystems, Foster City, CA, USA). The predicted cycle threshold (Ct) values were exported into Excel worksheets for analysis. The comparative Ct method with normalization to GAPDH was used to determine gene expression levels. Sequences of oligonucleotides used as primers were as follows: HMGCS2, forward, 5′-AAGTCTCTGGCTCGCCTGATGT-3′, reverse, 5′-TCCAGGTCCTTGTTGGTGTAGG-3′. E-cadherin, forward, 5′-GCAGTGACGAATGTGGTA-3′, reverse, 5′-GCTGTGGAGGTGGTGAGA-3′. Snail, forward, 5′-GCTGCAGGACTCAATCCAGA-3′, reverse, 5′-ATCTCCGGAGGTGGGATG-3′. Slug, forward, 5′-TGGTTGCTTCAAGGACACAT-3′, reverse, 5′-GTTGCAGTGAGGGCAAGAA-3′. Twist, forward, 5′-CCCAACTCCCAGACACCTC-3′, reverse, 5′-CAAAAAGAAAGCGCCCAAC-3’. Vimentin, forward, 5′-TGAACGCAAAGTGGAATC-3′, reverse, 5′-GTCAGGCTTGGAAACATC-3′. N-cadherin, forward, 5′-GGTGGAGGAGAAGAAGACCAG-3′, reverse, 5′-GCATCAGGCTCCACAGT-3′. Bax, forward, 5′-GATCCAGGATCGAGCAGA-3′, reverse, 5′- AAGTAGAAGAGGGCAACCAC-3′. Bcl-2, forward, 5′-AGGAAGTGAACATTTCGGTGAC-3’, reverse, 5′-GCTCAGTTCCAGGACCAGGC-3′. cyclin D1, forward, 5′-AGGAACAGAAGTGCGAGGAGG-3′, reverse, 5′-GGATGGAGTTGTCGGTGTAGATG-3′. GAPDH, forward, 5′-TCACCACCATGGAGAAGGC-3′, reverse, 5′-GCTAAGCAGTTGGTGGTGCA-3′.

### 4.10. Subcutaneous Xenograft Model

NOD/SCID mice, aged 7–8 weeks, were purchased from the Taiwan National Laboratory Animal Center. All mice were maintained on a standard chow diet (no. 5001, LabDiet, St. Louis, MO) or a ketogenic diet (5TJQ, TestDiet) and housed on a 12-h light–dark cycle. Since subcutaneous xenograft model is the most commonly used implantation model in the study of testing the changes in gene expression of HCC [56,57], Hep3B (shlacZ and shHMGCS2) and Huh-7 (eGFP and HMGCS2) cells (5 × 10^6^) were injected subcutaneously into both flanks of the NOD/SCID mice. Tumor growth was monitored three times a week by using Vernier caliper to measure of the length (L) and width (W) of the tumors. The tumor volume (TV) was calculated as follows: TV = (L × W^2^)/2. To alleviate and minimize potential pain, suffering, or distress in mice, when the tumor volume reached 1 cm^3^ in the vehicle control mice, monitoring was stopped. The protocol was reviewed and approved by the Institutional Animal Care and Use Committee of Taipei Medical University (LAC-2019-0186). Tumors and liver tissues were harvested at the end of the experiments. The samples used in protein and RNA analyses were frozen in liquid nitrogen and stored at −80 ℃, while those used for IHC staining were fixed in 10% formalin.

### 4.11. β-Hydroxybutyrate Treatment

Hep3B and Huh-7 shHMGCS2 cells (3 × 10^4^ cells/well) were seeded in 96-well plates, and after the cells were attached, medium containing 5, 10, or 20 mM βHB (Sigma-Aldrich, St. Louis, MO, USA) was added and incubated for the indicated time periods. Hepatic ketogenesis produces only D-βHB; thus, in this study, we focused on investigating the role of D-βHB.

### 4.12. Gene Expression Profiling

The mRNA profiles were analyzed using a Human OneArray Plus (Phalanx Biotech, Hsinchu, Taiwan). Total RNA was extracted from Huh-7 shlacZ and Huh-7 shHMGCS2 cells. Whole-genome gene expression was measured in these samples using OneArray Plus chips (Phalanx Biotech Group). Hierarchical clustering was performed using Cluster 3.0 (http://bonsai.hgc.jp/~mdehoon/software/cluster/). The differential expression of genes listed in the hierarchical clustering map was defined by the ratio of the expression in shHMGCS2 cells to that in shlacZ control cells as a log2 (fold change) of ≥2 (upregulation) or ≤0.5 (downregulation). The gene expression patterns in different pathways were analyzed using the KEGG pathway database (https://www.genome.jp/kegg/pathway.html).

### 4.13. Statistical Analysis

The results are expressed as the means ± standard deviations (SDs). The data were analyzed by nonparametric tests in SPSS v20.0 software (SPSS Inc., Chicago, IL, USA). The Mann–Whitney U test was used to compare two independent groups. Differences were considered statistically significant at *p* < 0.05.

## 5. Conclusions

Our study used a human liver disease tissue microarray, cellular experiments, and animal models to demonstrate that HMGCS2 controls the proliferation and migration abilities of liver cancer cells by regulating the apoptosis, c-Myc/cyclin D1, and EMT signaling pathways and acts in a ketogenesis enzyme-dependent manner. Taken together, our data suggest that HMGCS2 could be considered a promising targetable biomarker in future therapeutic interventions in HCC patients.

## Supplementary Materials

The following are available online at https://www.mdpi.com/2072-6694/11/12/1876/s1, Figure S1. Original unedited pictures and protein quantification of Figure 2. Figure S2. Investigation of cell morphology by light microscopy. Figure S3. KEGG enrichment pathway: TGF-β signaling pathway. Figure S4. KEGG enrichment pathway: tight junction pathway. Figure S5. KEGG enrichment pathway: pathway in cancer. Figure S6. IPA enrichment pathway: ERK/MAPK signaling. Figure S7. IPA enrichment pathway: PI3K/AKT signaling. Figure S8. IPA enrichment pathway: EMT pathway. Figure S9: IPA enrichment pathway: molecular mechanism of cancer. Figure S10. Original unedited pictures and protein quantification of Figure 3B. Figure S11 Original unedited pictures and protein quantification of Figure 3E. Figure S12. Original unedited pictures and protein quantification of Figure 4. Figure S13. Original unedited pictures and protein quantification of Figure 5.
