# Supplementary material for: HMGCS2 Mediates Ketone Production and Regulates the Proliferation and Metastasis of Hepatocellular Carcinoma
Source: Cancers (Basel). 2019 Nov 26;11(12):1876. doi: 10.3390/cancers11121876 (PMC6966636; doi:10.3390/cancers11121876)
Supplement: Supplementary file 1 [file cancers-11-01876-s001.pdf]

cancers

MDPI

Article

HMGCS2 Mediates Ketone Production and Regulates the Proliferation and Metastasis of Hepatocellular Carcinoma

Yuan-Hsi Wang [1,2], Chao-Lien Liu [1], Wan-Chun Chiu [3,4], Yuh-Ching Twu [2], and Yi-Jen Liao [1,*]

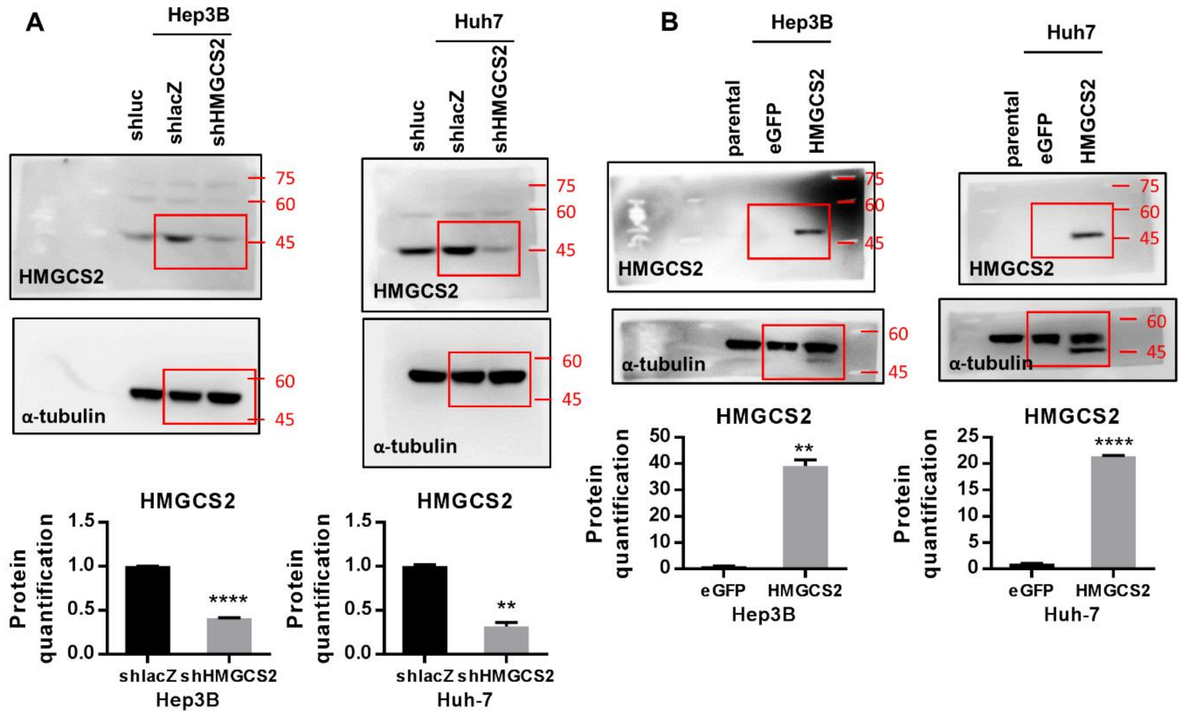

Figure S1. Original unedited pictures and protein quantification of Figure 2. Complete western blot image corresponding to Figure 2 showing all bands and molecular weight markers for HMGCS2 and α-tubulin. The quantification of the figure was presented by using ImageJ system. ** $p < 0.01$; **** $p < 0.0001$ vs. control group.

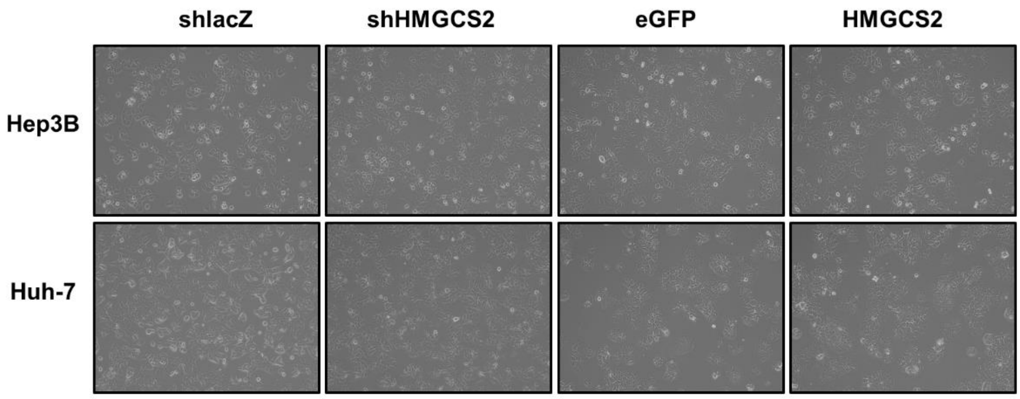

Figure S2. Investigation of cell morphology by light microscopy. Optical microscopy images in different HMGCS2 gene modified Hep3B and Huh-7 cells.

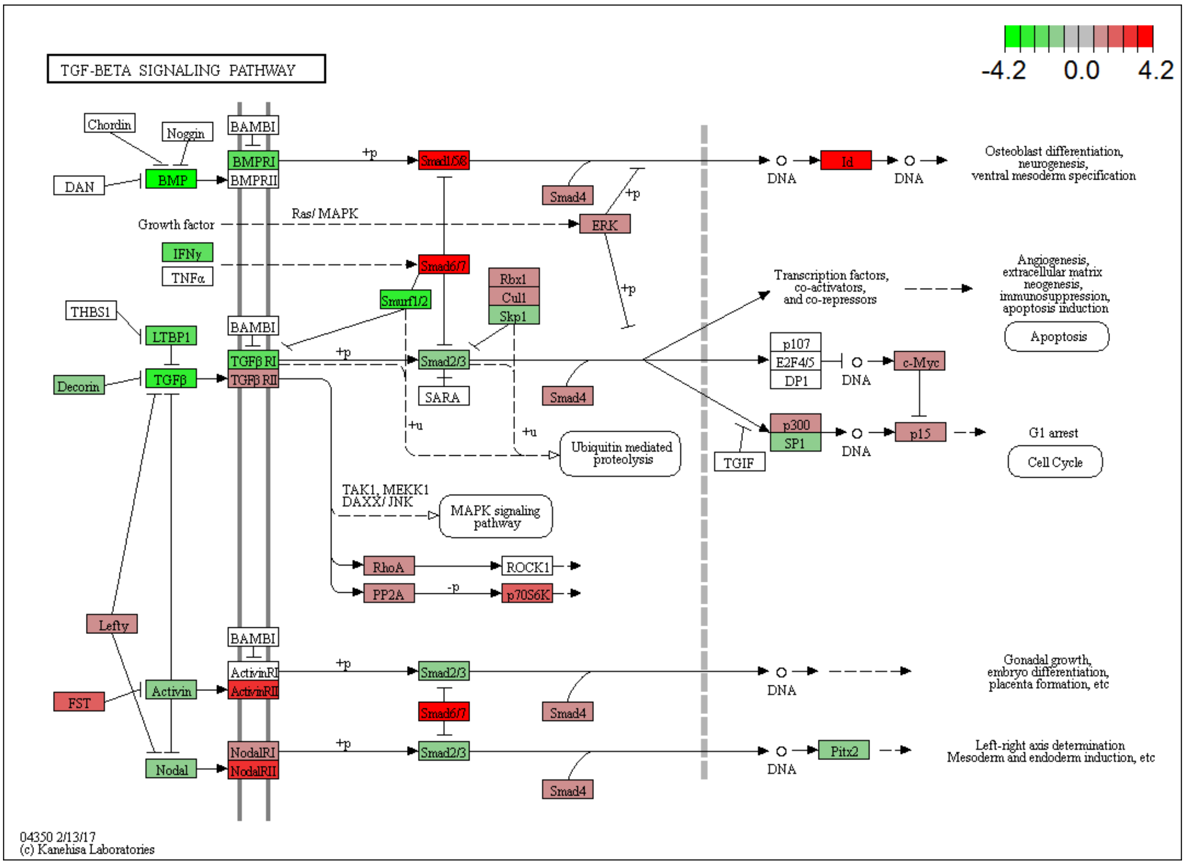

Figure S3. KEGG enrichment pathway: TGF-β signaling pathway. The down-regulation (green color shaded genes) and up-regulation (red color shaded genes) of most genes in this pathway in Huh-7 shlacZ vs. Huh-7 shHMGCS2 cells.

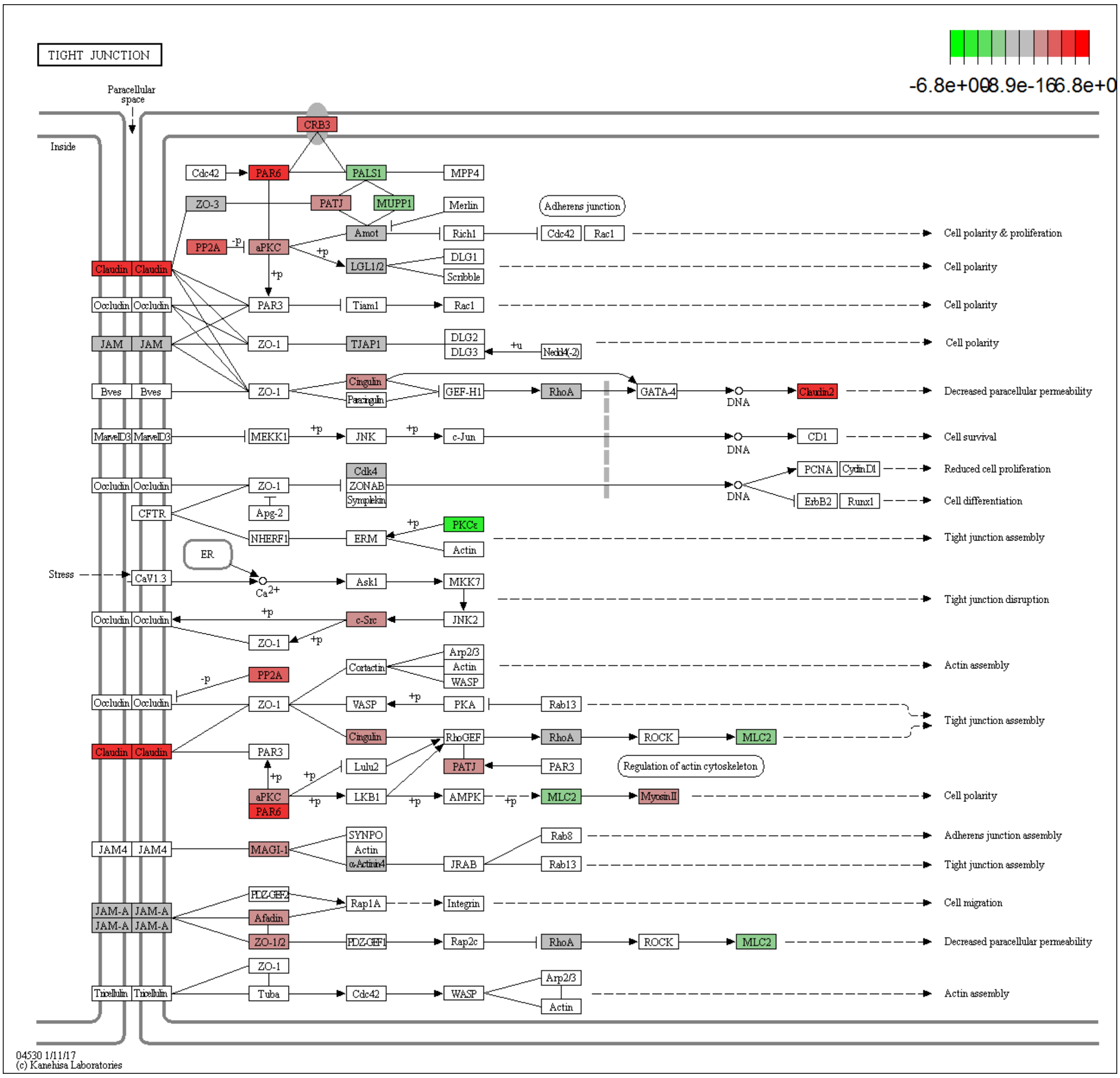

Figure S4. KEGG enrichment pathway: tight junction pathway. The down-regulation (green color shaded genes) and up-regulation (red color shaded genes) of most genes in this pathway in Huh-7 shlacZ vs. Huh-7 shHMGCS2 cells.

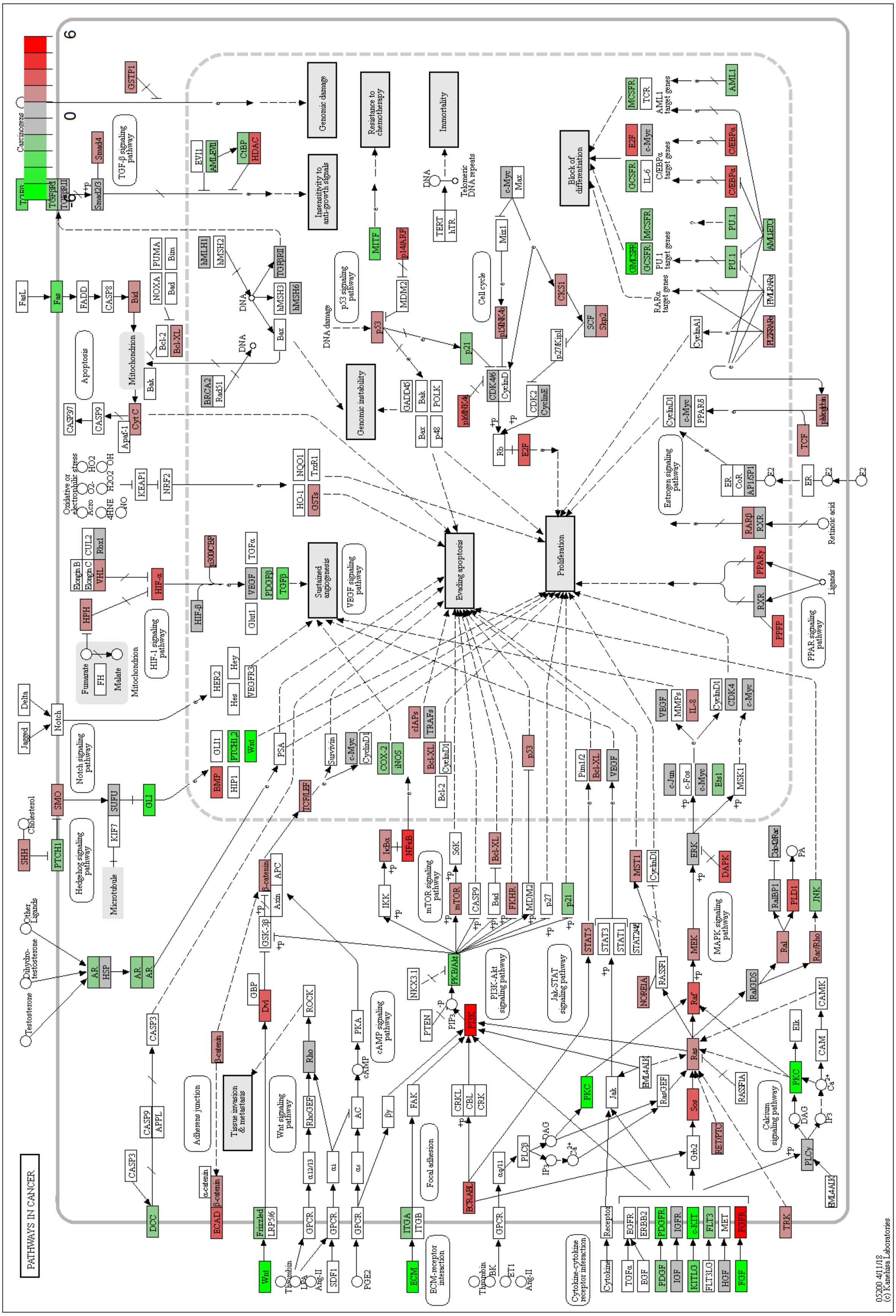

Figure S5. KEGG enrichment pathway: pathways in cancer. The down-regulation (green color shaded genes) and up-regulation (red color shaded genes) of most genes in this pathway in Huh-7 shlacZ vs. Huh-7 shHMGCS2 cells.

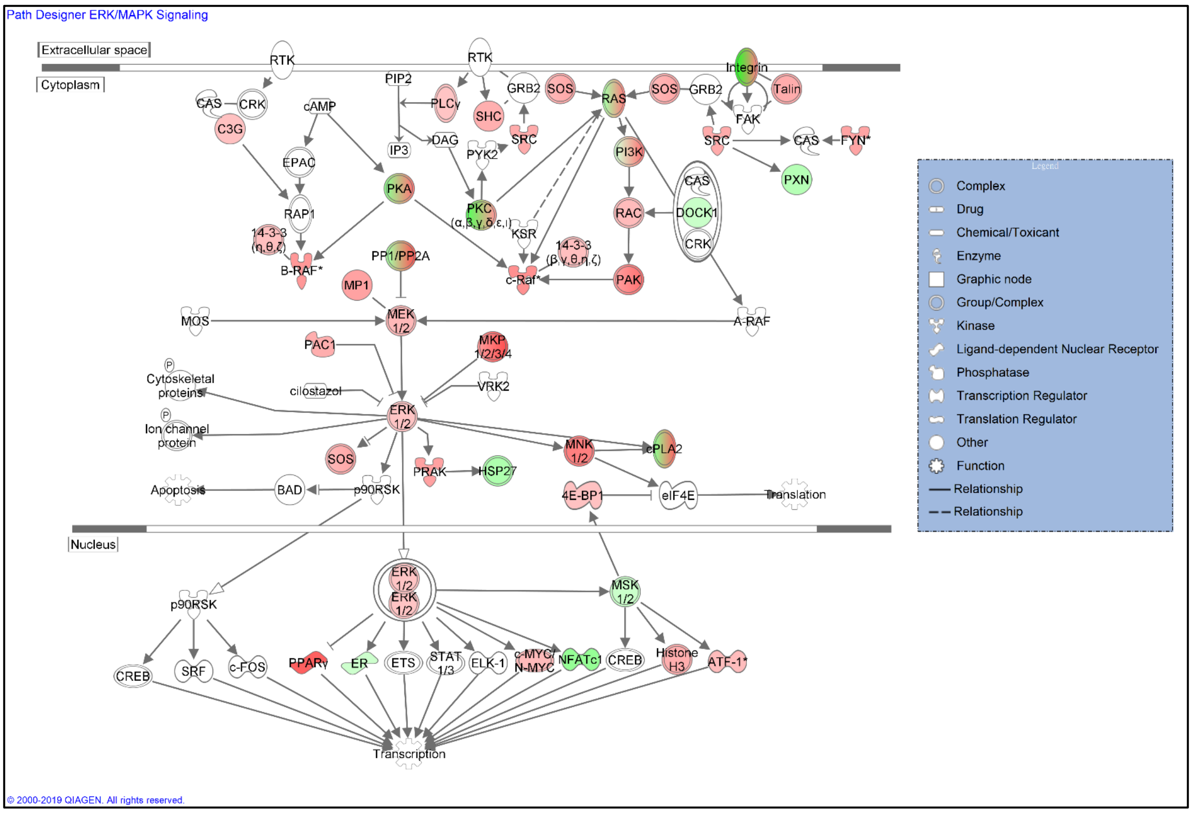

Figure S6. IPA enrichment pathway: ERK/MAPK signaling. The down-regulation (green color shaded genes) and up-regulation (red color shaded genes) of most genes in this pathway in Huh-7 shlacZ vs. Huh-7 shHMGCS2 cells.

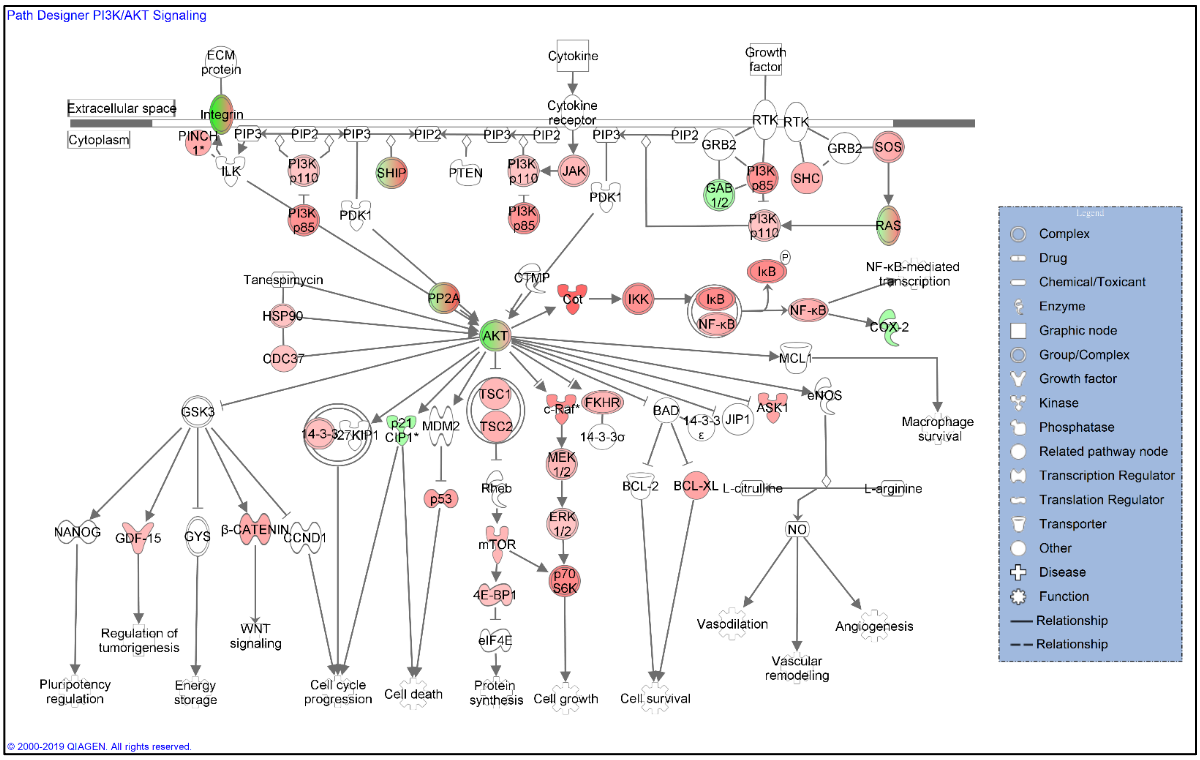

Figure S7. IPA enrichment pathway: PI3K/AKT signaling. The down-regulation (green color shaded genes) and up-regulation (red color shaded genes) of most genes in this pathway in Huh-7 shlacZ vs. Huh-7 shHMGCS2 cells.

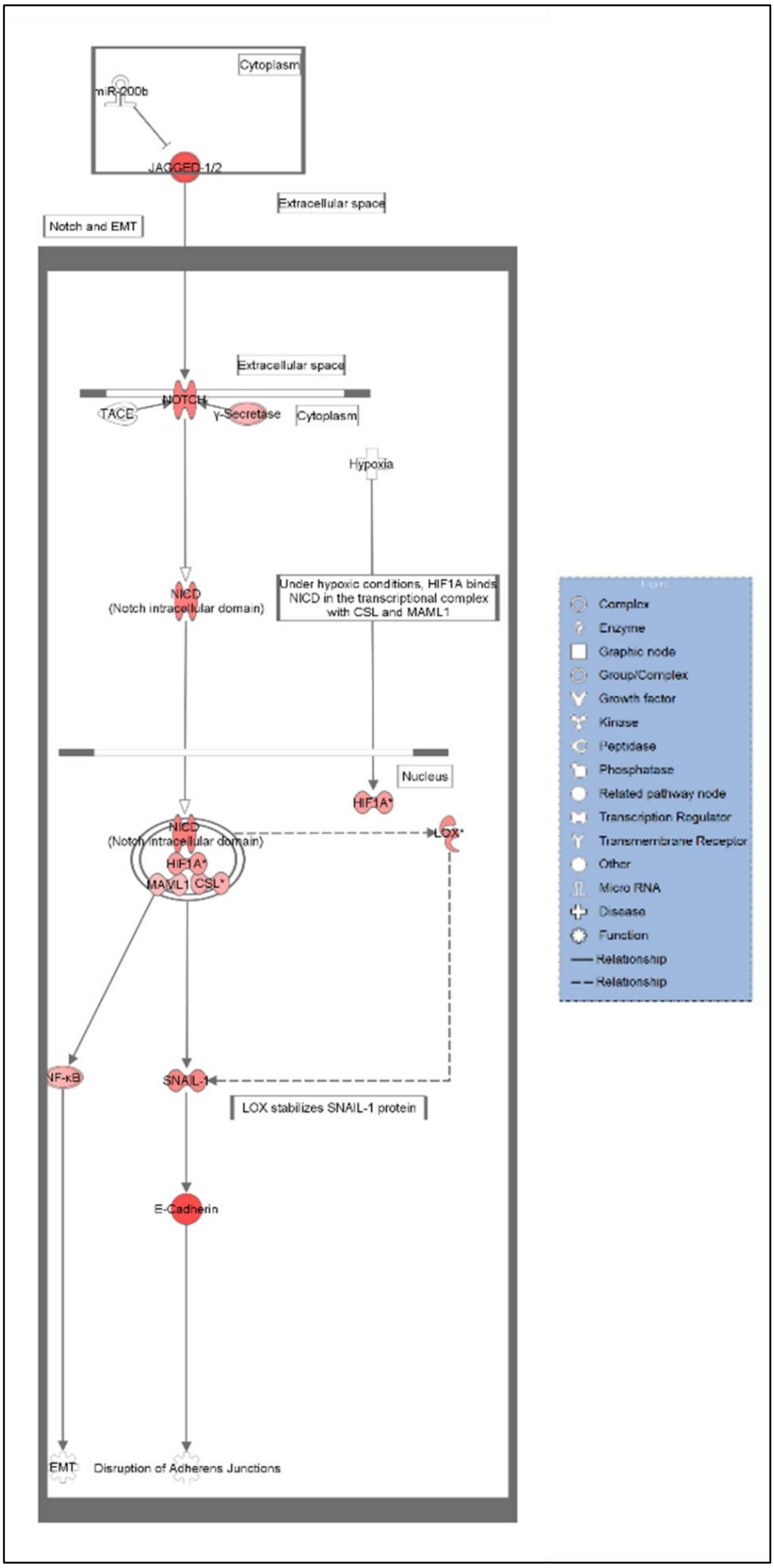

Figure S8. IPA enrichment pathway: EMT pathway. The down-regulation (green color shaded genes) and up-regulation (red color shaded genes) of most genes in this pathway in Huh-7 shlacZ vs. Huh-7 shHMGCS2 cells.

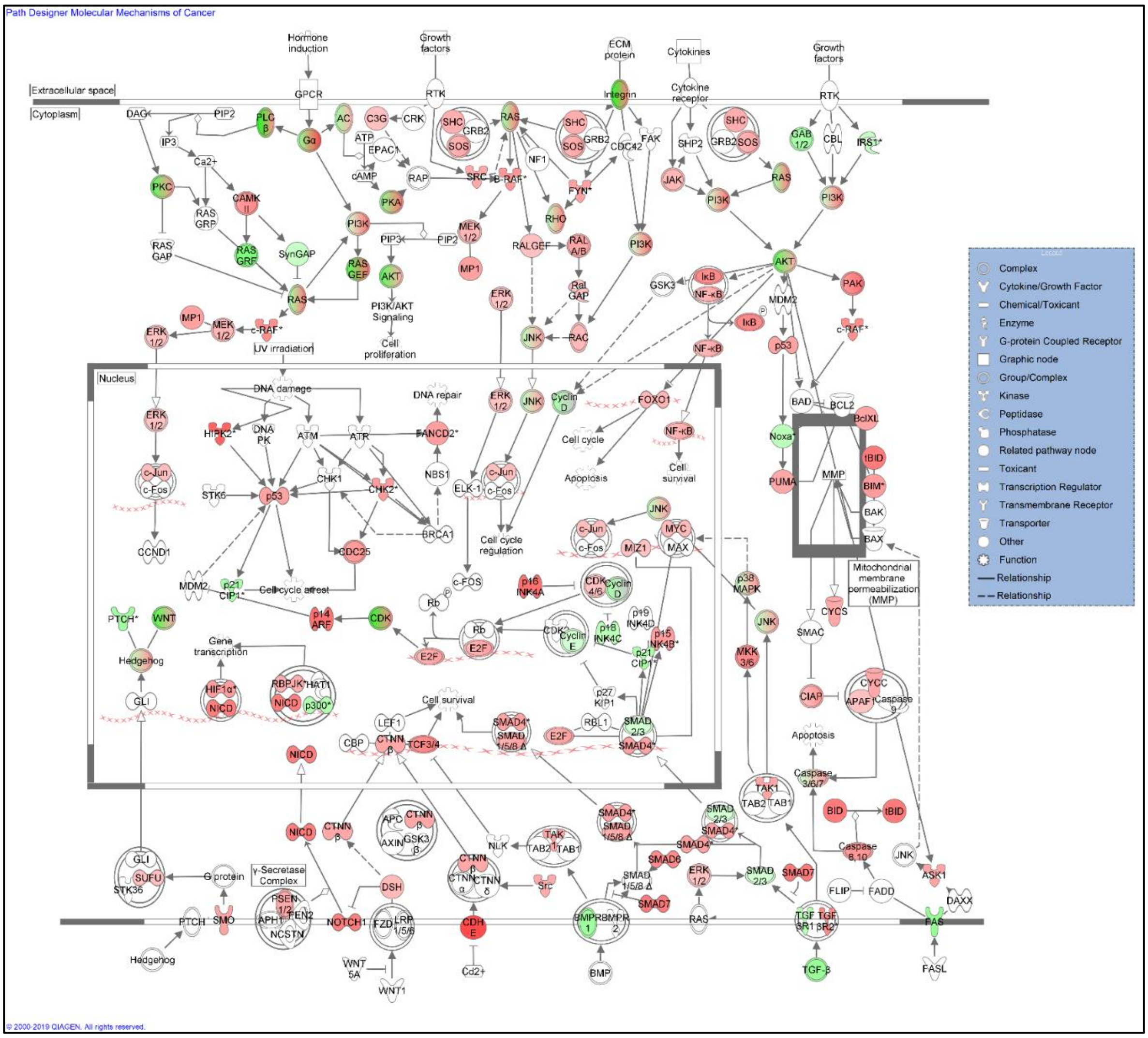

Figure S9. IPA enrichment pathway: molecular mechanism of cancer. The down-regulation (green color shaded genes) and up-regulation (red color shaded genes) of most genes in this pathway in Huh-7 shlacZ vs. Huh-7 shHMGCS2 cells.

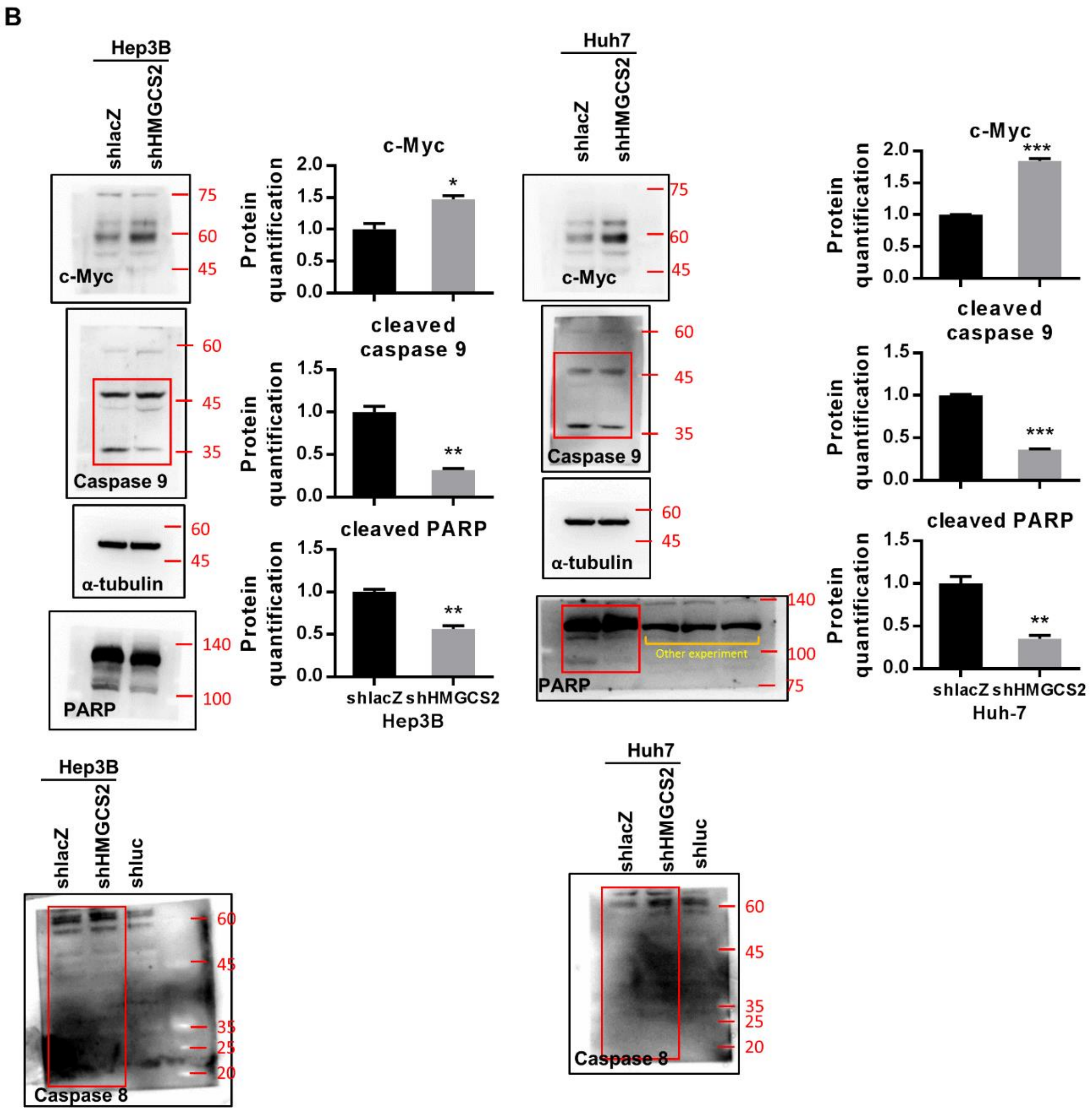

Figure S10. Original unedited pictures and protein quantification of Figure 3B. Complete western blot image corresponding to Figure 3B showing all bands and molecular weight markers for c-Myc, caspase9, caspase8, PARP, and α-tubulin. The quantification of the figure was presented by using ImageJ system. * $p < 0.05$; ** $p < 0.01$; *** $p < 0.001$ vs. control group.

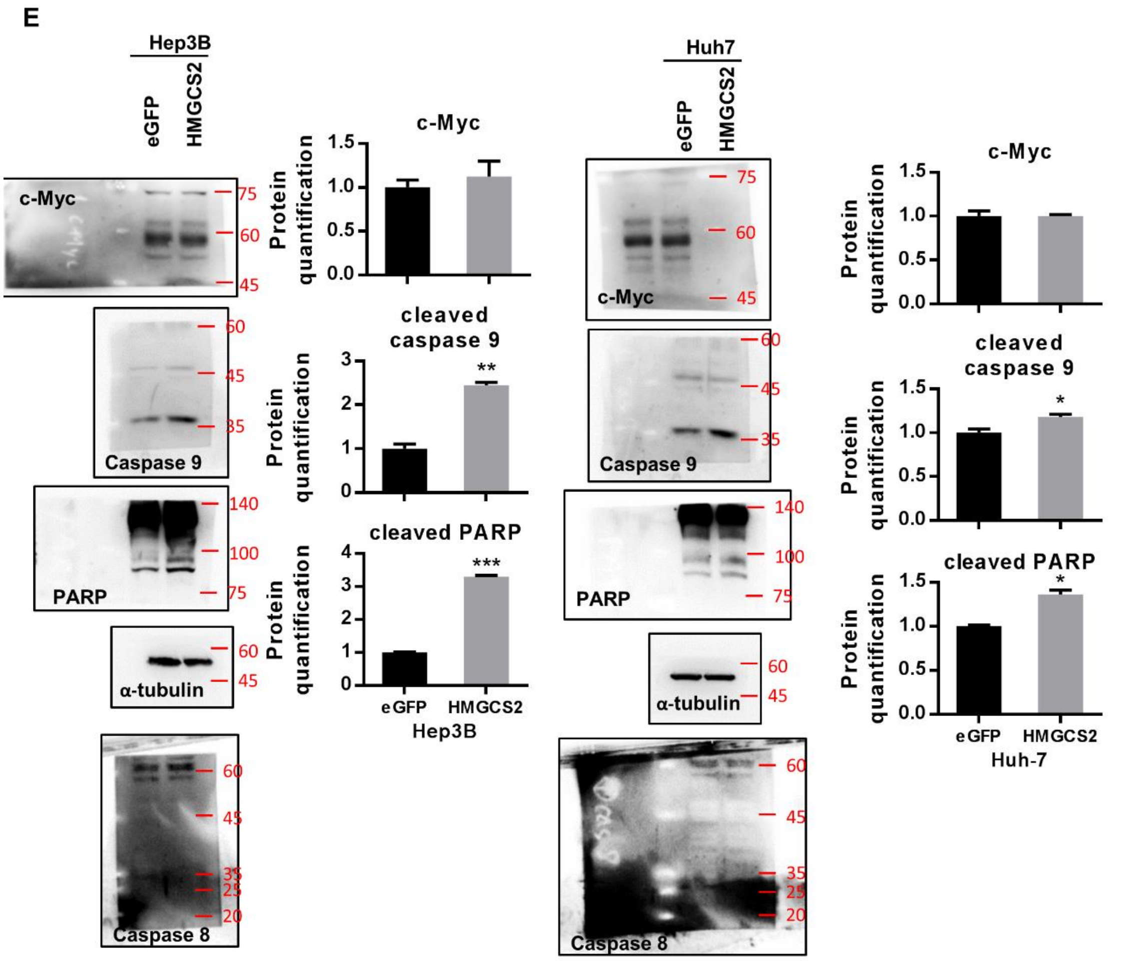

Figure S11. Original unedited pictures and protein quantification of Figure 3E. Complete western blot image corresponding to Figure 3E showing all bands and molecular weight markers for c-Myc, caspase9, caspase8, PARP, and α-tubulin. The quantification of the figure was presented by using ImageJ system. * $p < 0.05$; ** $p < 0.01$; *** $p < 0.001$ vs. control group.

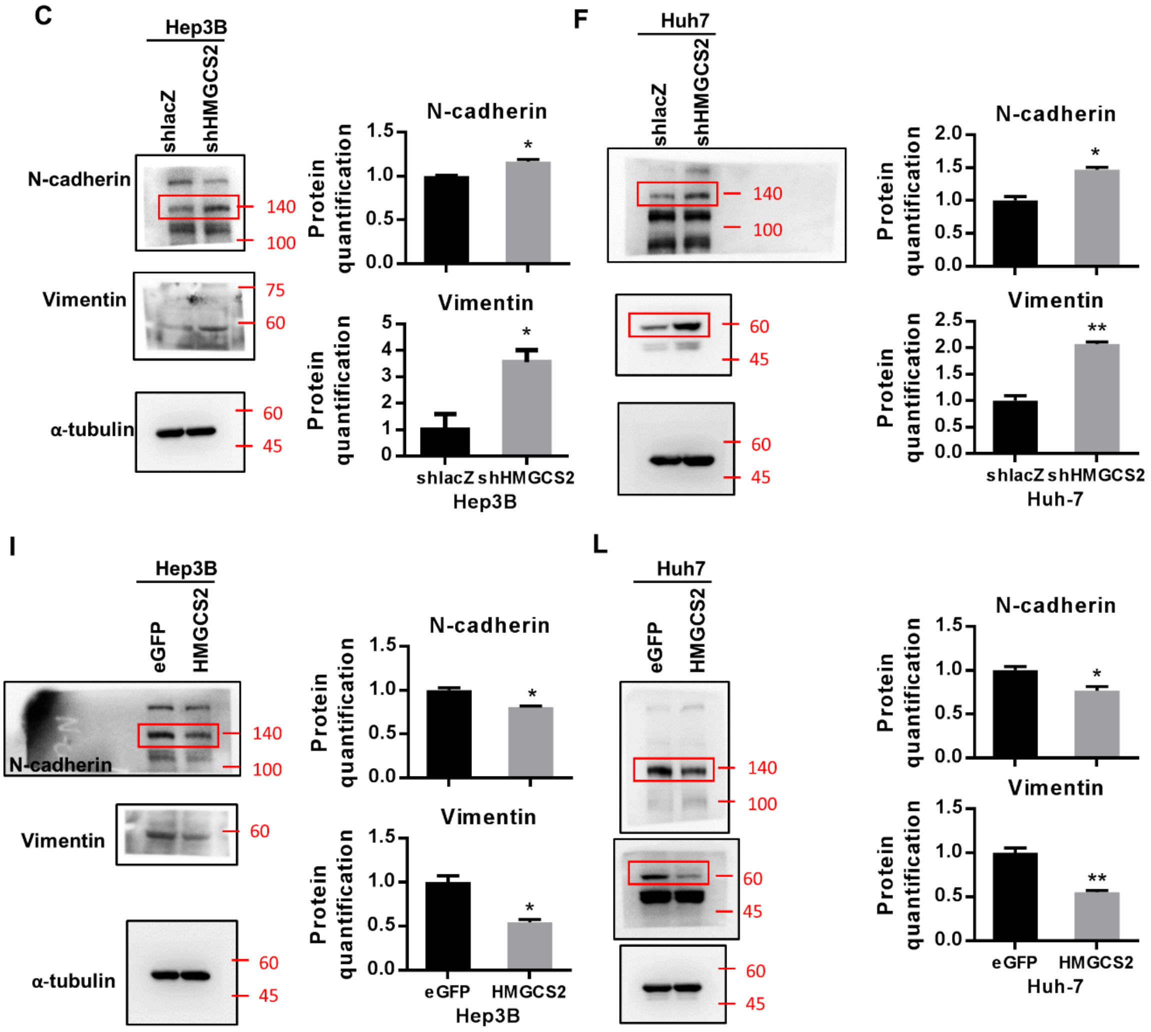

Figure S12. Original unedited pictures and protein quantification of Figure 4. Complete western blot image corresponding to Figure 4 showing all bands and molecular weight markers for N-cadherin, Vimentin, and α-tubulin. The quantification of the figure was presented by using ImageJ system. * $p < 0.05$; ** $p < 0.01$ vs. control group.

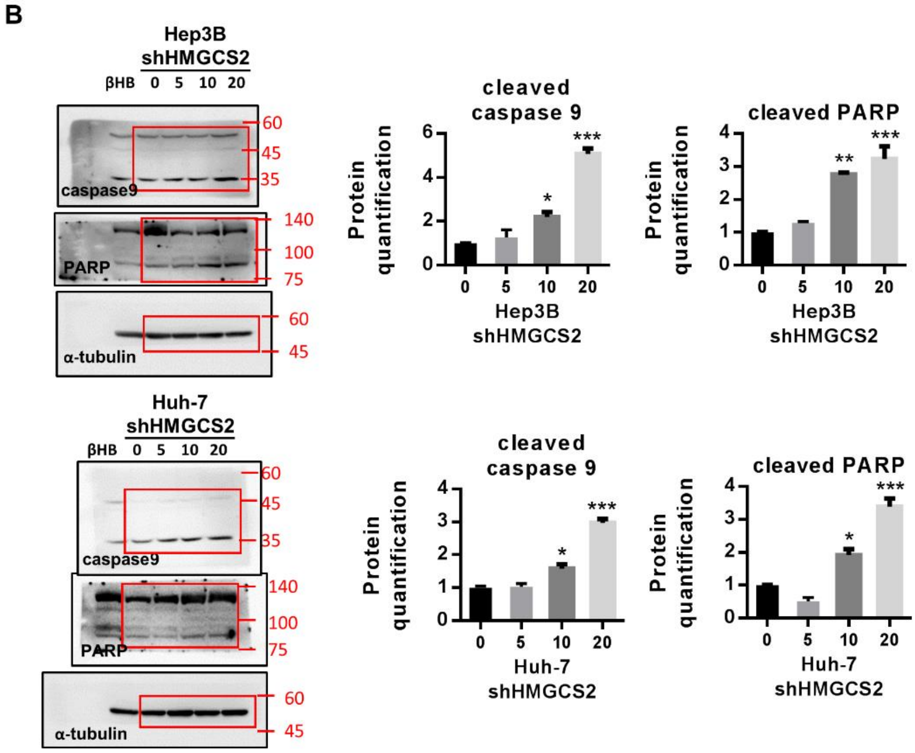

Figure S13. Original unedited pictures and protein quantification of Figure 5. Complete western blot image corresponding to Figure 5 showing all bands and molecular weight markers for caspase9, PARP, and α-tubulin. The quantification of the figure was presented by using ImageJ system.

© 2019 by the authors. Submitted for possible open access publication under the terms and conditions of the Creative Commons Attribution (CC BY) license (http://creativecommons.org/licenses/by/4.0/).
